# Genome-Wide Identification and Expression Analysis of the HD-Zip Gene Family in Wheat (*Triticum aestivum* L.)

**DOI:** 10.3390/genes9020070

**Published:** 2018-02-01

**Authors:** Hong Yue, Duntao Shu, Meng Wang, Guangwei Xing, Haoshuang Zhan, Xianghong Du, Weining Song, Xiaojun Nie

**Affiliations:** 1 College of Agronomy, State Key Laboratory of Crop Stress Biology in Arid Areas, Northwest A&F University, Yangling 712100, China; yuehongsx@163.com (H.Y.); wm2008xs@163.com (M.W.); xinggw920520@gmail.com (G.X.); zhanhaoshuang@nwsuaf.edu.cn (H.Z.); xianghongdu@nwsuaf.edu.cn (X.D.); sweining2002@yahoo.com (W.S.); 2 College of Life Sciences, Northwest A&F University, Yangling 712100, China; sqrs@163.com

**Keywords:** HD-Zip, gene family, wheat, abiotic stress, expression profiles

## Abstract

The homeodomain-leucine zipper (HD-Zip) gene family, as plant-specific transcription factors, plays an important role in plant development and growth as well as in the response to diverse stresses. Although HD-Zip genes have been extensively studied in many plants, they had not yet been studied in wheat, especially those involved in response to abiotic stresses. In this study, 46 wheat HD-Zip genes were identified using a genome-wide search method. Phylogenetic analysis classified these genes into four groups, numbered 4, 5, 17 and 20 respectively. In total, only three genes with A, B and D homoeologous copies were identified. Furthermore, the gene interaction networks found that the *TaHDZ* genes played a critical role in the regulatory pathway of organ development and osmotic stress. Finally, the expression profiles of the wheat HD-Zips in different tissues and under various abiotic stresses were investigated using the available RNA sequencing (RNA-Seq) data and then validated by quantitative real-time polymerase chain reaction (qRT-PCR) to obtain the tissue-specific and stress-responsive candidates. This study systematically identifies the HD-Zip gene family in wheat at the genome-wide level, providing important candidates for further functional analysis and contributing to the better understanding of the molecular basis of development and stress tolerance in wheat.

## 1. Introduction

The homeodomain-leucine zipper (HD-Zip) gene family is one of the key transcription factors in plants, playing a vital role in various abiotic stresses and signal transduction [1,2,3]. Generally, HD-Zip proteins possess the conserved HD domain, acting as a specific DNA binding site at the C-terminal, together with the adjacent leucine-zipper (LZ) motif that is responsible for protein dimerization [4,5]. Based on the sequence homology, DNA binding specificity and physiological functioning, HD-Zip genes were further divided into four groups, namely HD-Zip I, II, III and IV [6]. Members of groups I and II interacted with similar DNA binding sites of the pseudo-palindromic sequence CAATNATTG [5,7]. However, the members of group II encoded an additional common CPSCE motif consisting of five conserved amino acids—Cys, Pro, Ser, Cys and Glu—which acted as a sensory domain to redox the cell state and were located downstream of the Zip domain [8,9]. Group III and IV contained steroidogenicacute regulatory protein-related lipid transfer (START) and START-associated domain (SAD) domains [10,11,12]. The difference between groups III and IV were mainly dependent on a specific MEKHLA domain located in the C-terminal that responds to oxygen redox and light signaling [13]. In addition, the members of group III recognized sequences GTAATG/CATTAC, whereas members of group IV recognized sequences TAAATGC/TA [12,14,15]. 

Previous studies have extensively demonstrated that the HD-Zip family plays an important role in regulating diverse developmental and physiological processes in plants [16,17,18]. HD-Zip I proteins were found to be involved in the control of plant growth and development and also regulated the response to abiotic stresses [19]. The *TaHDZip I-2* gene regulated flowering and spike development and improved frost tolerance in transgenic barley lines [20]. In *Arabidopsis*, *AtHB1* acted downstream of *AtPIF1* to promote hypocotyl elongation, especially in response to short-day photoperiods [21], as well as mediated the leaf cell fate determination [22]. In cotton, the *GhHB1* expression level significantly increased in the early developmental roots, then significantly decreased as the roots developed, suggesting it might function in early root development [23]. Additionally, *TaHDZipI*-*3*, -*4* and -*5* genes were found to show the differential expression when wheat subjected to abscisic acid (ABA)treatment, cold and water deficit through binding the specific cis-elements [24].

HD-Zip II proteins regulated auxin signaling, participated in embryonic apical development and responded to light and abiotic stresses [25,26]. In *Arabidopsis*, some HD-Zip II proteins, including *AtHAT1*, *AtHAT2*, *AtHAT3*, *AtHB4* and *AtHB2*, regulated gene expression to adjust apical embryo development and meristem function [27]. *HaHB10* induced specific flowering transition gene expression in the transition stage from the vegetative to the flowering stage and induced accumulation of phytohormones in Arabidopsis under biotic stresses [28]. Additional evidences showed that the *EgHOX1* gene in oil palm was up-regulated by exogenous auxin and down-regulated by light [29]. HD-Zip III proteins were reported to impact vascular development, shoot and apical meristem formation, morph-physiological changes in roots and auxin transport [9,30]. C*LV3* and HD-Zip III pathways distinctively regulated meristem activity [31]. *KANADI* interacted with HD-Zip III genes to control lateral root development [32]. *PtrHB7* gene in *Populus* played an important role in balancing secondary growth in xylem cells and phloem tissues [33]. Additionally, some studies have revealed that HD-Zip III genes could be negatively regulated by *MicroRNA165/166* [33,34]. HD-Zip IV proteins were the regulators for trichome development, epidermal cell differentiation and root formation [35]. *PDF2* played a vital role in the epidermis cells to adjust normal development of the floral organs [36]. *OCL4* inhibited trichome development in *Arabidopsis* and maize and influenced anther cell division and differentiation [37]. In cucumber, *CsGL3* and *CsGL1* genes regulated trichome initiation and development [38]. 

Wheat is one of the most important cereal crops in the world, occupying 17% of cultivated lands and serving as the staple food source for 30% of the human population [39]. Abiotic stresses are the main limiting factor for wheat production worldwide, which negatively impact wheat growth and development, resulting in huge yield losses. Between the 2000 and 2008, wheat grain production fell by 5.5% annually due to adverse climates [40]. To improve the stress tolerance of wheat, identification and use of the elite gene resource may help meet the challenges of the changing global climate. Although some studies have studied the function of wheat HD-Zip genes [20,41,42], their genome organization, structure and evolutionary features are not well-understood, especially those involved in the regulatory processes of abiotic stresses. In this study, we systematically characterized the HD-Zip genes in wheat using the latest genome sequences. The genome composition, phylogeny, conserved motifs, chromosome localization, regulatory network and the expression profiles of TaHDZ proteins were systematically analyzed, providing a basis for further investigation of the functions of *TaHDZ* genes. The results will help reveal the molecular mechanism of development and stresses response in wheat and other cereal crops.

## 2. Materials and Methods

### 2.1. Genome-Wide Identification of HD-Zip Gene Family in Wheat

Wheat genome and protein sequences (release 36, accessed in 2017) were obtained from the Ensemble plants database [43] to predict HD-Zip genes. These sequences were first used to construct a local protein database with which to search against known HD-Zip protein sequences collected from *A. thaliana* (48) and *O. sativa* (48), through a local protein basic local alignment search (BLASTP) program (https://blast.ncbi.nlm.nih.gov) with an E-value cut-off <10^−5^ and an identity of 50% as the threshold. The hidden Markov model (HMM) profile of the conserved HD domain of homeobox (PF00046) and the leucine zipper (LZ) domain (PF02183) sequences were download from the PFAM database [44] and used to examine all wheat protein sequences using the HMMER search tool [45]. After manual curating, the obtained protein sequences were checked using theNational Center for Biotechnology Information (NCBI)–Conserved domain database (CDD) search [46] to identify the conserved protein domain with the default parameters. The redundant sequences containing complete HD and LZ domains were further removed by alignment and the remaining ones was considered as putative wheat HD-Zip genes (*TaHDZ*). The coding sequences of these HD-Zip genes were retrieved from the wheat genome annotation information. Finally, Compute pI/MW tool in ExPASy database [47] was used to calculate the biochemical parameters of *TaHDZ*s. Sub-cellular localization of these genes was predicted by Cell-Ploctool software [48].

### 2.2. Phylogenetic Analysis and Gene Duplication

The *Arabidopsis* HD-Zip proteins were downloaded from the *Arabidopsis* information resource (TAIR) database (http://web.arabidopsis.org) and those of rice were obtained from the rice genome annotation project (http://rice.plantbiology.msu.edu/). To investigate the evolutionary relationships among these *TaHDZ* genes, the ClutsalX1.83 program [49] was used to align the protein sequences of the wheat, rice and *Arabidopsis* HD-Zip genes (Supplementary file 1). The phylogenetic tree was constructed using MEGA6.0) [50] with the neighbor-joining (NJ) method and 1000 bootstrap replications.

The chromosome localizations of these genes were analyzed by mapping the gene sequences back to chromosome using the nucleotide basic local alignment search (BLASTN) program (https://blast.ncbi.nlm.nih.gov) with the E-value cutoff <10^−5^ and the best hits were identified. Gene duplication was investigated following the method as described by Wang et al. [51]. Based on chromosome position and phylogenetic relationship, the homoeologous copies distributed in three sub-genomes A, B or D of wheat were identified. Then, the Circos tool [52] was used to visualize the duplicated regions in the wheat genome and the same colors show the homoeologous chromosomal segments.

### 2.3. Gene Structure and Protein Conserved Motifs Analysis

The gene structure information was obtained from the Ensemble plants database [43] and displayed in the gene structure display server (GSDS) program [53]. Conserved motifs of these genes were determined using the multiple EM for motif elicitation (MEME) program [54] with the parameters as follow: optimum motif widths of 6–200 residues and a maximum of 20 motifs. The schematic diagram of the amino acid motifs for each *TaHDZ* gene was drawn accordingly.

### 2.4. Expression Profile Analysis of TaHDZ Genes

The publicly available wheat RNA-Seq datasets were downloaded from the URGI database [55] and the NCBI sequence read archive (SRA) database (https://www.ncbi.nlm.nih.gov/sra), then used to analyze the expression profiles of the identified *TaHDZ* genes (Table S2). A total of 5 tissues including root, stem, leaf, spike and grain, as well as four stress treatments including cold (SRR1460552), heat (SRR1542413), drought (SRR1542409) and salt (SRR2306546) were used to identify tissue-specific or stress-responsive ones. Evaluation of the quality of RNA-Seq reads and trimming of the low-quality readings with a Phred quality (Q) score < 20 were performed using FastQCv0.11.5 [56]. Then, TopHat version 2.1.1 [57] was used to map the RNA-Seq reads to the wheat genome (release v36). Cufflinks Version 2.2.1 [58] was used to calculate the value of fragments per kilo base of transcript per million fragments mapped (FPKM) of these genes with the default parameters. The expression level was first normalized and then the log_10_-transformed values were used for visualizing the heat map using the R software (https://www.r-project.org/).

### 2.5. Interaction Network of TaHDZ Genes

To predict the regulatory role of *TaHDZ* genes, the interaction networks of *TaHDZ*s with other wheat genes was constructed based on the orthologous relationship between *Arabidopsis* and wheat using the AraNetV2 tool [59]. The *Arabidopsis* gene ontology (GO) biological processes were used as the major reference set. The Cytoscape plugin, BiNGO [60], was used to analyze the orthologous gene and identify the biological pathways of the specific gene sets.

### 2.6. Plant Materials, Growth Conditions and Abiotic Stress Treatments

Seeds were planted in pots and filled with 1/2 Hoagland’s liquid medium and grown in a greenhouse at 22 °C, with a 16 h photoperiod (12,000 lux) and 8 h dark period. Wheat variety Dekang No. 685 (DK, salt-tolerant) and Chinese spring (CS, salt-susceptible) were used for the salt treatment. Besides, Hanxuan No. 10 (HX, drought-tolerant) and CS were used for the drought treatment. For salt stress, two-week seedlings of DK and CS were treated with 200 mM sodium chloride (NaCl) for 24 h. HX and CS were treated with 19.2% polyethylene glycol (PEG) for 24 h to represent the drought treatment. Then, the plants materials were collected and immediately frozen in liquid nitrogen for RNA extraction. All samples were replicated three times.

### 2.7. Quantitative Real-Time Polymerase Chain Reaction Analysis

cTotal RNA was isolated from all the collected samples using the Plant RNA isolation kit (OmegaBioTek, Norcross, GA, USA), following the manufacturer’s instructions. The RNA quality was checked using 1.0% (*w/v*) agarose gel stained with ethidium bromide (EB) and spectrophotometer analysis and then DNase I treatment was conducted to remove the DNA contaminations (Takara, Shiga-ken, Japan). The first strand complementary DNA (cDNA)s were synthesized using the cDNA amplification kit (Vazyme, Nanjing, China). The *TaHDZ* gene primers and 18S, as the reference gene, were used for quantitative real-time polymerase chain reaction (qRT-PCR), designed using Primer Premier 5.0 software [61] and are listed in Table S1. Then, qRT-PCR was performed using CFX96Touch (Bio-Rad, Hercules, CA, USA). Three biological replicates for each sample were performed and the expression level was evaluated using the 2^−∆∆Ct^ method. 

## 3. Results and Discussion

### 3.1. Identification of TaHDZ Genes in Wheat

The availability of the complete genome sequence allowed the identification and analysis of gene family at the genome level in wheat. Increasing numbers of gene families such as MAPKKK, Aux/IAA and Annexin have been reported in wheat [34,62,63]. HD-Zip, as one of the plant-specific transcription factor gene families, is vital for regulating plant development and physiological processes as well as in response to abiotic stresses. However, little is known about the HD-Zip gene family in wheat. We identified and characterized the wheat HD-Zip gene family based on a genome-wide search approach. A total of 46 non-redundant genes containing the complete HD and LZ domains were obtained, which were considered as the putative wheat HD-Zip genes. These TaHDZ proteins ranged in length from 100 to 883 amino acids, with molecular weights ranging from 14.62 kDa to 95.73 kDa and the isoelectric points ranged from 4.61 to 10.68.

Subcellular localization analysis indicated that 21 *TaHDZ* are localized in the nucleus, 20 in the chloroplast, whereas only three and two were found in the peroxisome and mitochondrion, respectively (Table 1). Moreover, the proteins were grouped into 28 clusters based on their phylogenetic relationship. Among them, 15 clusters were assigned to different A, B or D sub-genomes, which were considered as the homoeologous copies of one *TaHDZ* gene. Finally, there are 28 clusters of HD-Zip gene family in wheat and termed as *TaHDZ1-A* to *TaHDZ28-D* according to their chromosomes position (Table 1). The size of HD-Zip family in wheat is similar to that of rice, maize and peal millet, as well as *Arabidopsis* with 48, 55, 52 and 48, respectively, while are much higher than that of grape (*Vitis vinifera* L.) with 31 members, chrysanthemum (*Chrysanthemum morifolium* L.) with 17 members and citrus (*Citrus sinensis* L.) with 27 members and much lower than that of soybean (*Glycine max* L.) with 88 members and poplar (*Populus trichocarpa* L.) with 65 members [9,64,65,66,67]. A previous study reported that the abundance of the HD-Zip gene family was not correlated to genome size, which was mainly dependent on tandem and segmental duplications during the process of genome evolution [65]. Our results show that the HD-Zip gene family did not expand during wheat polyploidization and genome evolution. Besides, fewer duplication events of HD-Zip genes were found in wheat. 

### 3.2. Chromosome Localization Analysis of TaHDZs

**Figure 1:**
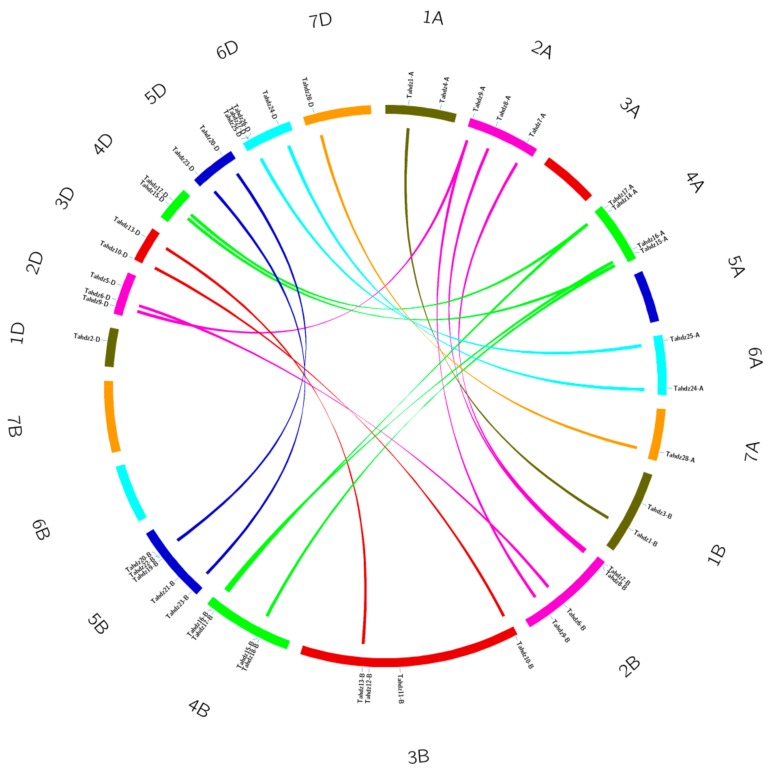
Chromosomal localization and gene duplication identified in wheat. Seven chromosomes of wheat A, B and D sub-genomes are displayed in different colors. Duplicated gene pairs are exhibited in linked lines with the corresponding color.

Chromosome distribution was further analyzed by BLASTN against wheat genome sequence. Results revealed that the HD-Zip genes were unevenly distributed on wheat chromosomes. In total, 12, 19 and 15 *TaHDZ* were located on the A, B and D sub-genome, respectively. Chromosome groups two and four had 10 HD-Zip genes each, representing the most abundant regions, followed by group five with seven. At the same time, no *TaHDZ* genes were found on chromosomes 3A, 5A, 6B, or 7B (Figure 1).

Gene duplication is generally the main factor causing the expansion of the given gene family [68]. As a genetically allohexaploid species, wheat has a complex origin and evolutionary history, derived from three diploid donor species through two naturally interspecific hybridization events. As aresult, each wheat gene generally has three homologous loci arising from polyploidization [69]. Though sequence similarity and chromosome localization analysis, three *TaHDZ* genes, including *TaHDZ9*, *TaHDZ15 andTaHDZ17*, were found to have three copies on each of the A, B and D homoeologous chromosome. Twelve *TaHDZ* genes (*TaHDZ1, -6, -7, -8, -10, -13, -16, -20, -23, -24, -25* and *-28*) contain two copies in the A, B, or D homoeologous chromosome. Just one copy of the remaining 13 genes was found in wheat chromosomes (Figure 1). Previous studies revealed that inversion and crossover might occur between homologous chromosomes during polyploidization to fractionate the coding region or delete some homologous sequences [70]. Our results indicated considerable homologous genes loss may occur in the wheat HD-Zip gene family, causing the loss of some homologous copies. The retention and dispersion of specific HD-Zip genes in homologous chromosomes could provide some insights into the mechanism of wheat chromosome interaction and genome evolution.

### 3.3. Phylogenetic Analysis of TaHDZ Genes

**Figure 2:**
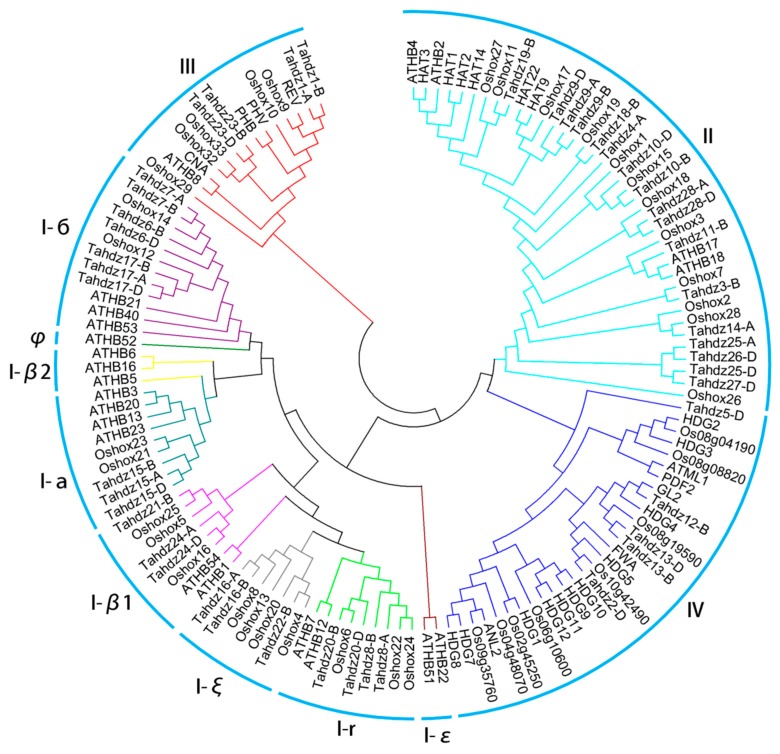
Phylogenetic analysis of HD-Zip proteins among wheat (46), *Arabidopsis* (48) and rice (39).

**Figure 3:**
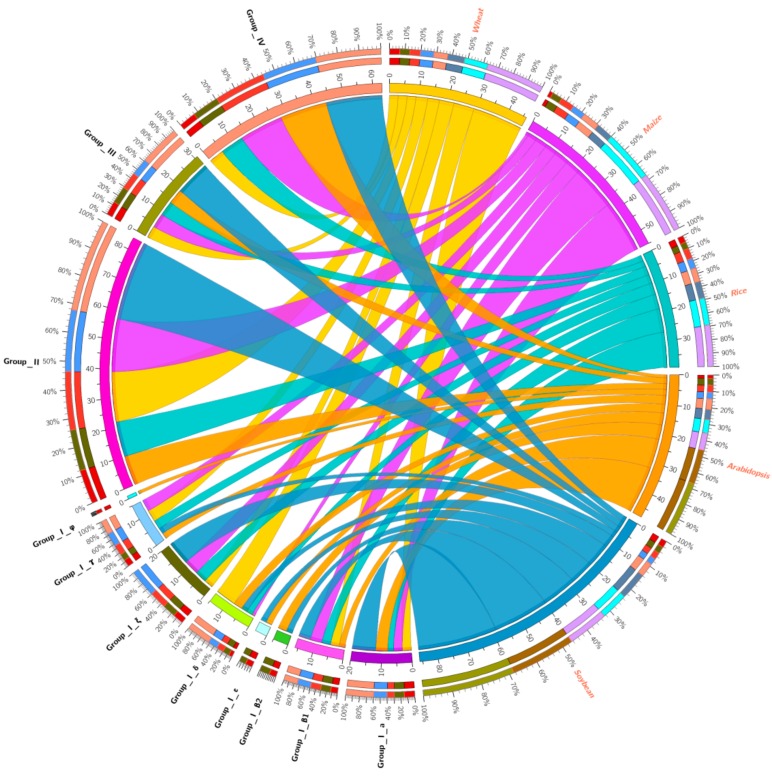
The distribution of HD-Zip transcription factors from wheat, maize, *Arabidopsis*, rice and soybean.

To investigate the phylogenetic relationships of the HD-Zip gene family, 46 TaHDZ proteins, together with 48 and 39 publicly available *Arabidopsis* and rice HDZ proteins, were selected for phylogenetic analysis. Based on the classification criteria in *Arabidopsis* and rice [6], the wheat HDZ proteins were clustered into four groups, I to IV. Specifically, 20, 17, 4 and 5 TaHDZ proteins were classified into groups I, II, III and IV, respectively. Group I was further divide into eight sub-groups, including group I-а (3), group I-β1 (3), group I-β2 (0), group I-б (7), group I-ε (0), group I-ξ (3), group I-r (4) and group I-φ (0) (Figure 2). Among them, group I was the most abundant in wheat, rice and *Arabidopsis*, accounting for 43%, 35% and 35%, respectively, similar to that of grape and sorghum having a percentage of 42% and 32%, respectively [9]. In contrast, in maize group II is the largest group, accounting for 32.72% [12,65]. Furthermore, no proteins were categorized into subgroup I-β2 and I-ε in wheat, maize, or rice but more than two members were present in both soybean and *Arabidopsis* (Table 2). The HD-Zip gene family distribution has been reported to be predominant with a species bias [9,12]. This result indicated that gene loss in these sub-groups might occur during the divergence of dicots and monocots. Additionally, the number of proteins in group I-б in wheat was significantly higher, with seven members, compared to one in maize, two in rice, three in *Arabidopsis* and two in soybean, suggesting some *TaHDZ* genes also expanded, although duplication events of HD-Zip genes were unusual in wheat (Table 2 and Figure 3).

### 3.4. Co-Expression Network between TaHDZ Genes and Other Genes in Wheat

**Figure 4:**
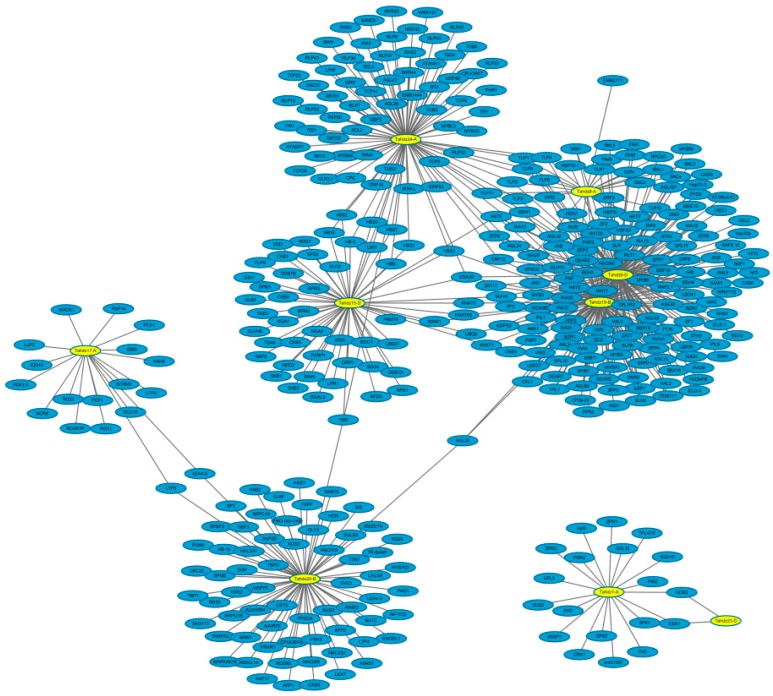
The co-expression network of *TaHDZ* genes in wheat according to the orthologues in *Arabidopsis*.

To identify the biological function and interaction relationship between *TaHDZ*s and other wheat genes, their interaction network was constructed using an orthology-based method. A total of nine *TaHDZ* genes were homologous with *Arabidopsis*, with 191 gene pairs of network interactions found, suggesting that TaHDZ proteins are widely involved in the wheat metabolic network and regulated diverse biological processes and pathways (Figure 4). Interacting genes were classified into three major gene ontology classes using GO annotations: diverse biological process, cellular component and molecular function (Table S2). Previous studies found that *AtHB7* is involved in the regulation of organ development and plays a critical role in the response to osmotic stress [71]. In this study, *TaHDZ20-B* was found as the orthologous gene of *AtHB7* in the wheat HD-Zip family. Further analysis found that they interacted with 71 wheat genes, including the stress-responsive gene *LEA*, *MAPKKK18*, *MYB* and *NAC*, suggesting they may be mainly involved in the response to abiotic stresses (Figure 4). The identified homology genes and the putative co-expression network analysis of *TaHDZ*s have provided useful information for the further study of the biological function and transduction pathways of HD-Zip genes in wheat.

### 3.5. Conserved Motifs and Expression Profile Analysis of TaHDZ Genes

**Figure 5:**
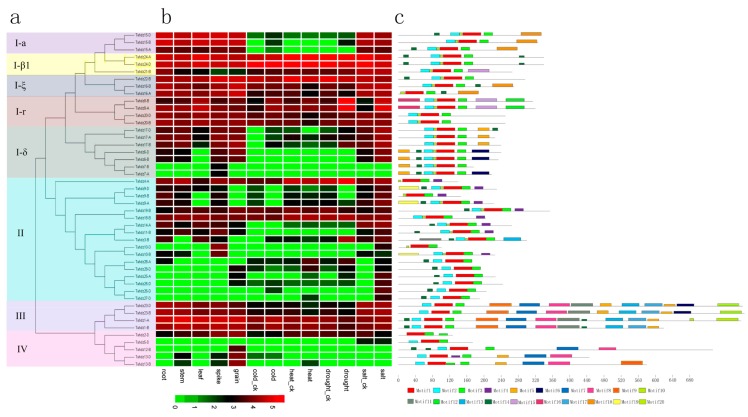
(**a**) Phylogenetic relationships; (**b**) Expression patterns; (**c**) Conserved motifs compositions of the 46 HD-Zip genes in wheat. (**a**) The phylogenetic tree was constructed based on the full-length protein sequences using MEGA6.0 (http://web.megasoftware.net/); (**b**) Hierarchical clustering of the relative expression level of *TaHDZ* genes. RNA-Seq data of five tissues and four stresses in Chinese spring was used to analysis expression pattern. The heat map was drawn in Log_10_-transformed expression values. Red or green colors represent decreased or increased expression level in each sample, respectively; (**c**) Multiple EM for motif elicitation (MEME) program [54] was used to predict conserved motifs. Each motif is represented by a different colored box.

Different members of gene families generally exhibit disparities in abundance in different tissues or with different stressors [72]. To gain insight about the putative functions of the *TaHDZ* genes, the temporal and spatial expression profile of these identified *TaHDZ* genes were examined using the publicly available RNA-Seq data (Figure 5). Results showed that some tissue-specific genes, such as *TaHDZ7-A/B* specifically expressed in the wheat spike, were found, although most members of group I had no significant expression differences in the five organs. Furthermore, the stress-responsive group I members were also analyzed. Result found that *TaHDZ15-A/B/D* were expressed in normal conditions, whereas higher expression was found after 6h of cold and drought stress. Additionally, the *TaHDZ8-A/B* transcript level increased for up to 6h after exposure to cold, heat, drought and salt stress (Figure 5b), suggesting they play a role in response to stress.

In group II, five genes, *TaHDZ10-B/D*, *TaHDZ25-A/D*, *TaHDZ28-A/D*, *TaHDZ3-B* and *TaHDZ9-A/B/D*, had significant expression differentiation in different tissues, whereas *TaHDZ25-A/D*, *TaHDZ26-D*, *TaHDZ27-D* and *TaHDZ28-A/D* showed significant high expression under stresses. Among them, *TaHDZ26-D* and *TaHDZ27-D* were markedly induced by cold and heat stress, respectively. The expression patterns of group I and II genes revealed that wheat HD-Zip I and II genes are relevant for a variety of stresses. Notably, the cold-responsive genes mainly belonged to group I, whereas the salt-responsive genes belonged to group II, consistent with previous results that indicated that group I and II genes play important roles in response to various stresses in plants [73]. For groups III and IV, most of the members showed no significant expression differences in different tissues or with different stresses (Figure 5b). However, *TaHDZ13-B/D*, belonging to group III, had a relatively high expression in the spike and grain but weak expression in the root and leaf, suggesting involvement in the reproduction process. *TaHDZ13-B/D* was the one member of group IV that showed significantly up-regulation under heat stress compared to the control, meaning it could be considered heat-responsive gene (Figure 5b).

To obtain insight into the relationship between gene structure and expression of *TaHDZ*s, the conserved motifs in these protein sequences were further predicted using the MEME program. A total of 20 conserved motifs were found (Figure 5c). The identified *TaHDZs* motifs ranged from 8 to 50 amino acids in length. The details of the sequence of all conserved motifs are shown in Figure S1. The motifs were unevenly distributed in those proteins, with the number of motifs ranging from 3 to 13. It showed that motif one and two, corresponding to the HD domain and motif three, corresponding to the LZ domain, were distributed in all the TaHDZ proteins. Notably, proteins in the same group seemed to share similar motif compositions. For examples, three motifs, including motif six, eight and nine were found to be related to the START domain, which was present in groups III and IV subfamilies, with the exception of *TaHDZ5-D*. Motif four, encoding the CPSCE domain, was present in each group II member, except for *TaHDZ25*, *-26*, *-27* and *-28*. Additionally, the gene structure might also be involved in the control of gene expression patterns in various tissues or abiotic stresses (Figure 5b,c). For examples, motif 11, 17 and 20 only existed in *TaHDZ23B/D* and *TaHDZ1A* and these genes had relatively high expression in various tissues or under abiotic stresses. However, group I-б and IV, containing motif five, had relatively low expression in various tissues or under abiotic stresses (Figure 5b,c and Figure S1).

### 3.6. Expression Profiles of TaHDZ Genes under Abiotic Stress in Wheat by qRT-PCR Analysis

**Figure 6:**
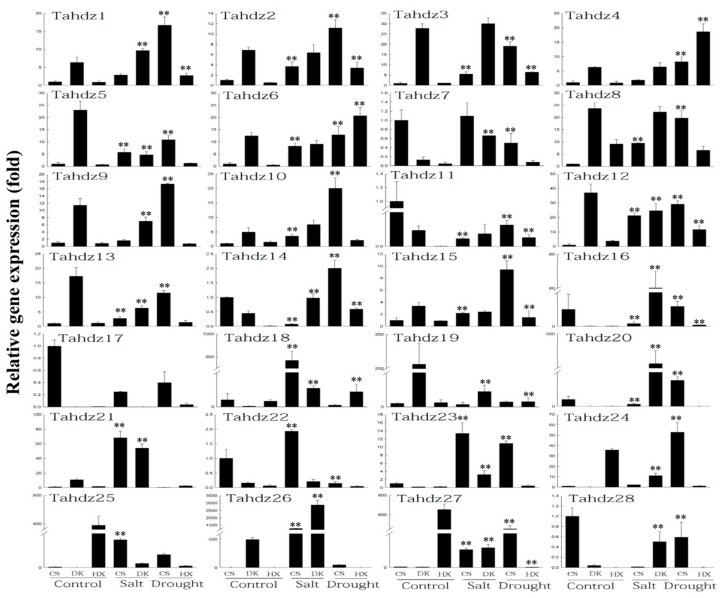
The expression profiles of 28 *TaHDZ* genes that may be involved in the response to salt or drought stress in different wheat varieties using quantitative real-time polymerase chain reaction (qRT-PCR) analysis. For salt stress, two-week-old seedlings of variety Dekang No. 685 (DK) and Chinese Spring (CS) were treated with 200 mM sodium chloride (NaCl) for 24 h. Hanxuan No. 10 (HX) and CS were treated with 19.2% polyethylene glycol (PEG) for 24 h to represent the drought treatment. The control was an untreated seedling. Three biological replicates for each sample were performed and bars represented the standard deviations of the mean. Asterisks on top of the bars indicating statistically significant differences between the stress and counterpart controls (** *p*< 0.01, Student’s *t*-test). Gene expression profiles were evaluated using the 2^−∆∆C^ method.

To validate the stress-responsive candidates, 28 *TaHDZ*s showing differential expression underabiotic stresses, based on the RNA-Seq data, were selected to conduct quantitative polymerase chain reaction (qPCR) analysis. Results showed that these genes had differential expression under different stresses and between tolerant and susceptible genotypes. In CS, which is susceptible to salt and drought stress, a total of 21 and 20 *TaHDZ* genes were induced by salt and drought stress, respectively, whereas 18 were induced by salt stress in the DK genotype (salt-tolerant) and 20 were induced by drought stress in the HX genotype (drought-tolerant). In total, 13 *TaHDZ* genes were induced by salt stress in both DK and CS. Among them, *TaHDZ11* and *TaHDZ19* were down-regulated and the others were up-regulated. Sixteen *TaHDZ* genes were induced by drought in both HX and CS, of which *TaHDZ11*, *TaHDZ17 andTaHDZ18* were rapidly reduced 0.06-fold, 0.39-fold and 0.26-fold under drought stress in CS, whereas these genes were strongly induced to 50-fold, 100-fold and 2.64-fold in HX under drought treatment, suggesting these genes might play a vital role in the response to drought stress in wheat (Figure 6).

A large number of HD-Zip genes have been demonstrated to regulate the abiotic stresses response in model plants [12,25,26,71], providing indications of the biological function of wheat HD-Zip genes using orthology-based predictions. *Oshox22* was found to be participate in ABA-mediated signal pathways, regulating drought and salt responses in rice [74]. Through phylogenetic analysis, *TaHDZ8* was found as the orthologous gene of *Oshox22* in this study. RNA-Seq data showed that *TaHDZ8* was only lightly expressed 6h after exposure to abiotic stress (Figure 5c). Using qRT-PCR, the results showed that the expression level of *TaHDZ8* in CS under salt or drought stress was 9.50-fold and 18.61-fold higher than those of the control, respectively. The expression level of *TaHDZ8* was 33.25-fold higher in DK and 8.13-fold higher in HX than in CS as the control (Figure 6). In addition, *AtHB7* and *AtHB12* were revealed to be involved in the fine-tuning processes associated with growth and drought stress [71]. Their orthologue counterpart in wheat—*TaHDZ20* and *TaHDZ28*—had significant differential expressions between control and drought stress in susceptible genotypes while no significant difference in tolerant genotype (Figure 6), suggesting these two genes may play the essential role in the regulatory network of drought response in wheat.

## 4. Conclusions

This study systematically identifies and characterizes the wheat HD-Zip gene family at the whole genome level. A total of 46 wheat HD-Zip genes, belonging to groups I to IV, were identified, and they were unevenly distributed on wheat chromosomes. The gene structure, conserved motif and phylogenetic relationship analysis further supported the classification. Gene duplication analysis found that 13, 12 and 3 *TaHDZs* were found to have one, two and three homoeologous copies, respectively, suggesting homoeologous gene loss events occurred in this family. Furthermore, the co-expression network between *TaHDZs* and other wheat genes was constructed. Up to 191 interactions could be found, indicating that TaHDZ proteins are widely involved in the wheat metabolic network and that they regulate diverse biological processes and pathways. Finally, the expression profiles of these *TaHDZ* genes in different tissues and under various stress conditions were identified by RNA-Seq mapping and further validated by qRT-PCR analysis. In total, thirteen salt-responsive and sixteen drought-responsive genes were obtained, which should be considered as the candidates for future abiotic functional studies. Overall, this study provides the genetic background knowledge for genetic improvement of salt and drought tolerance in wheat, and contributes as well to reveal the molecular mechanism of stress response.

## Figures and Tables

**Table 1:** Characteristics of the putative homeodomain-leucine zipper (HD-Zip) proteins in wheat.

Gene	Sequence ID	Location	AA Length	PI	MW	Subcellular Location
*TaHDZ1-A*	Traes_1AL_0BE456AC0.1	1A:80794504-80805037	840	5.65	92,041.09	Chloroplast
*TaHDZ1-B*	Traes_1BL_43408C9B0.2	1B:202405337-202411629	620	5.80	68,027.69	Chloroplast
*TaHDZ2-D*	Traes_1DL_9FB53E48A.1	1DL:scaff527273:1-682	128	9.00	14,621.61	Nucleus
*TaHDZ3-B*	Traes_1BL_BCA60D8B6.2	1BL:scaff3858366:4943-6233	302	6.68	33,291.33	Nucleus
*TaHDZ4-A*	Traes_1AL_1444D461A.1	1A:192016994-192017547	326	8.50	34,647.92	Nucleus
*TaHDZ5-D*	Traes_2DL_036F2A3FC.1	2DL:scaff9746565:6-712	251	8.31	26,889.50	Chloroplast
*TaHDZ6-B*	Traes_2BS_BD0ED621D.2	2B:203096624-203098080	238	7.12	25,888.92	Chloroplast
*TaHDZ6-D*	Traes_2DS_20F748657.2	2D:47948060-47949499	238	6.76	25,755.72	Chloroplast
*TaHDZ7-A*	Traes_2AL_CC3E5591E.1	2A:214032194-214033814	218	8.84	24,308.27	Peroxisome
*TaHDZ7-B*	Traes_2BL_419CEED79.1	2BL:scaff8047670:2819-3802	196	9.54	21,615.02	Nucleus
*TaHDZ8-A*	Traes_2AL_BFB0C6D4C.1	2A:92585473-92586697	183	5.15	20,209.38	Chloroplast
*TaHDZ8-B*	Traes_2BL_B69300543.1	2BL:scaff8082479:11478-12572	259	5.20	29,003.43	Nucleus
*TaHDZ9-A*	Traes_2AL_EF9549D16.1	2AL:scaff6334009:1565-3326	227	8.84	25,675.08	Nucleus
*TaHDZ9-B*	Traes_2BL_02479C76A.1	2B:265061413-265062697	159	9.78	18,432.80	Nucleus
*TaHDZ9-D*	Traes_2DL_67F1183B2.1	2D:25397187-25398979	230	8.84	25,914.27	Nucleus
*TaHDZ10-B*	TRAES3BF043500070CFD_t1	3B:8112566-8113594	228	8.64	25,282.62	Nucleus
*TaHDZ10-D*	Traes_3DS_7CCB5ECD2.1	3D:813820-814506	183	9.12	20,819.69	Nucleus
*TaHDZ11-B*	TRAES3BF026400090CFD_t1	3B:424323223-424326762	222	9.42	24,704.13	Nucleus
*TaHDZ12-B*	TRAES3BF023000040CFD_t1	3B:530484425-530487959	755	7.80	81,462.58	Chloroplast
*TaHDZ13-B*	TRAES3BF075200070CFD_t1	3B:554475326-554479704	674	8.48	73,671.85	Chloroplast
*TaHDZ13-D*	Traes_3DL_8AAFB7B06.1	3D:86091404-86095021	446	8.33	49,134.62	Nucleus
*TaHDZ14-A*	Traes_4AL_822582A19.1	4AL:scaff7079911:4919-6438	266	9.26	28,237.92	Chloroplast
*TaHDZ15-A*	Traes_4AS_F04DD4409.1	4AS:scaff5975837:1-1802	278	6.76	28,363.81	Chloroplast
*TaHDZ15-B*	Traes_4BL_BE3E058A6.1	4BL:scaff7026111:366-3006	325	9.24	33,237.48	Nucleus
*TaHDZ15-D*	Traes_4DL_88ABAD6C0.1	4DL:scaff14448085:2765-5409	330	6.17	36,411.50	Nucleus
*TaHDZ16-A*	Traes_4AL_99A941299.1	4A:184972869-184975111	318	4.94	34,975.56	Mitochondrion
*TaHDZ16-B*	Traes_4BL_ECD20BE67.1	4B:292827483-292829670	316	4.99	35,099.79	Nucleus
*TaHDZ17-A*	Traes_4AS_1EA23DE08.1	4AS:scaff3077305:961-2471	231	6.24	25,530.54	Peroxisome
*TaHDZ17-B*	Traes_4BL_BE10705D5.2	4B:282781983-282783490	233	6.04	25,576.61	Peroxisome
*TaHDZ17-D*	Traes_4DL_4798D0BBD.1	4D:68945470-68946969	234	6.24	25,708.82	Chloroplast
*TaHDZ18-B*	Traes_4BL_78DD63002.1	4BL:scaff6966681:8297-10341	205	8.94	22,631.64	Chloroplast
*TaHDZ19-B*	Traes_5BL_4A3874701.1	5B:170563162-170564690	355	6.27	37,049.33	Nucleus
*TaHDZ20-B*	Traes_5BL_9C32B27E2.1	5BL:scaff10897212:246-2341	249	5.02	27,502.62	Nucleus
*TaHDZ20-D*	Traes_5DL_96F9EED93.2	5DL:scaff4539911:3583-5740	249	5.02	27,488.59	Nucleus
*TaHDZ21-B*	Traes_5BL_028D02DF6.1	5B:84457851-84460016	299	4.86	32,566.01	Chloroplast
*TaHDZ22-B*	Traes_5BL_5DE02D63E.1	5BL:scaff10833801:1080-2780	269	4.70	28,884.88	Chloroplast
*TaHDZ23-B*	Traes_5BS_360DD5644.1	5B:18562939-18569007	879	6.61	95,339.96	Chloroplast
*TaHDZ23-D*	Traes_5DS_50846FD0C.1	5D:21777479-21783627	883	6.73	95,733.49	Chloroplast
*TaHDZ24-A*	Traes_6AL_36AB0312C.1	6A:185224197-185226370	340	4.61	37,072.67	Chloroplast
*TaHDZ24-D*	Traes_6DL_FF4C8C4AB.1	6D:138591996-138594281	340	4.67	37,147.85	Chloroplast
*TaHDZ25-A*	Traes_6AS_3E534A2C1.1	6AS:scaff4406943:3994-5166	226	8.72	24,538.57	Chloroplast
*TaHDZ25-D*	Traes_6DS_17B737547.1	6D:24274468-24275712	225	8.38	24,759.74	Nucleus
*TaHDZ26-D*	Traes_6DS_D281B7D32.1	6D:23048583-23049661	205	9.87	22,606.56	Nucleus
*TaHDZ27-D*	Traes_6DS_F00EB2E01.1	6D:21598812-21599534	192	9.79	20,888.56	Chloroplast
*TaHDZ28-A*	Traes_7AL_44206BE21.1	7A:149156319-149157237	169	10.68	19,423.03	Mitochondrion
*TaHDZ28-D*	Traes_7DL_2FE5181AF.1	7DL:scaff1534355:1875-3077	185	9.50	20,757.18	Nucleus

ID: identity; AA: amino acids; PI: isoelectric point; MW: molecular weight.

**Table 2:** The number of HD-Zip genes in wheat, maize, *Arabidopsis*, rice and soybean.

Species	Group I-a	Group I-β1	Group I-β2	Group I-ε	Group I-б	Group I-ξ	Group I-r	Group I-φ	Group II	Group III	Group IV	Total
wheat	3	3	0	0	7	3	4	0	17	4	5	46
Maize	3	4	0	0	1	5	4	0	18	5	15	55
Rice	2	3	0	0	2	4	3	0	12	5	8	39
Arabidopsis	4	2	3	2	3	0	2	1	10	5	16	48
Soybean	8	4	2	2	2	8	2	0	27	12	19	86

## Supplementary Materials

The following are available online at http://www.mdpi.com/2073-4425/9/2/70/s1, Figure S1: Sequence logos of TaHDZ proteins conserved motifs. A total of 20 motifs were identified using the MEME tool, Table S1: Primer sequences of 28 *TaHDZ* genes used for qRT-PCR analysis, Table S2: Details of 9 *TaHDZ* orthologous genes between wheat and *Arabidopsis thaliana*, Supplementary file 1: The multiple sequence alignment of TaHDZ proteins.

## References

[1] Hu W., Wang L.Z., Tie W.W., Yan Y., Ding Z.H., Liu J.H., Li M.Y., Ming P., Xu B.Y., Jin Z.Q. (2016). Genome-wide analyses of the bZip family reveal their involvement in the development, ripening and abiotic stress response in banana. Sci. Rep..

[2] Chan R.L., Gago G.M., Palena C.M., Gonzalez D.H. (1998). Homeoboxes in plant development. Biochim. Biophys. Acta.

[3] Mcginnis W., Garber R.L., Wirz J., Kuroiwa A., Gehring W.J. (1984). A homologous protein-coding sequence in *Drosophila* homeotic genes and its conservation in other metazoans. Cell.

[4] Ruberti I., Giovanna S., Lucchetti S., Morelli G. (1991). A novel class of plant proteins containing a homeodomain with a closely linked leucine zipper motif. EMBO J..

[5] Wolfgang F., Phillips J., Salamini F., Bartels D. (1998). Two dehydration-inducible transcripts from the resurrection plant *Craterostigma plantagineumencode* interacting homeodomain-leucine zipper proteins. Plant J..

[6] Ariel F.D., Manavella P.A., Dezar C.A., Chan R.L. (2007). The true story of the HD-Zip family. Trends Plant Sci..

[7] Meijer A.H., Scarpella E., van Dijk E.L., Qin L., Taal A.J., Rueb S., Harrington S.E., McCouch S.R., Schilperoort R.A., Hoge J.H. (1997). Transcriptional repression by Oshox1, a novel homeodomain leucine zipper protein from rice. Plant J..

[8] Tron A.E., Bertoncini C.W., Chan R.L., Gonzalez D.H. (2002). Redox regulation of plant homeodomain transcription factors. J. Biol. Chem..

[9] Hu R., Chi X., Chai G., Kong Y., He G., Wang X., Shi D., Zhang D., Zhou G. (2012). Genome-wide identification, evolutionary expansion and expression profile of homeodomain-leucine zipper gene family in poplar (*Populus trichocarpa*). PLoS ONE.

[10] Ponting C.P., Aravind L. (1999). START: A lipid-binding domain in StAR, HD-ZIP and signalling proteins. Trends Biochem. Sci..

[11] Schrick K., Nguyen D., Karlowski W.M., Mayer K.F. (2004). START lipid/sterol-binding domains are amplified in plants and are predominantly associated with homeodomain transcription factors. Genome Biol..

[12] Chen X., Zhu C., Zhao H.L., Zhao Y., Cheng B.J., Xiang Y. (2014). Genome-wide analysis of soybean HD-ZIP gene family and expression profiling under salinity and drought treatments. PLoS ONE.

[13] Mukherjee K., Bürglin T.R. (2006). MEKHLA, a novel domain with similarity to PAS domains, is fused to plant homeodomain-leucine zipper III proteins. Plant Physiol..

[14] Sessa G., Steindler C., Morelli G., Ruberti I. (1998). The *Arabidopsis* Athb-8,-9 and genes are members of a small gene family coding for highly related HD-ZIP proteins. Plant Mol. Biol..

[15] Abe M., Katsumata H., Komeda Y., Takahashi T. (2003). regulation of shoot epidermal cell differentiation by a pair of homeodomain proteins in *Arabidopsis*. Development.

[16] Di Cristina M., Sessa G., Dolan L., Linstead P., Baima S., Ruberti I., Morelli G. (1996). The *Arabidopsis* Athb-10 (GLABRA2) is an HD-Zip protein required for regulation of root hair development. Plant J..

[17] Kubo H., Peeters A.J., Aarts M.G., Pereira A., Koornneef M. (1999). *ANTHOCYANINLESS*2, a homeobox gene affecting anthocyanin distribution and root development in *Arabidopsis*. Plant Cell..

[18] Yuan D., Tang Z., Wang M., Gao W., Tu L., Jin X., Chen L., He Y., Zhang L., Zhu L. (2015). the genome sequence of Sea-Island cotton (*Gossypium barbadense*) provides insights into the allopolyploidization and development of superior spinnablefibres. Sci. Rep..

[19] Mao H., Yu L., Li Z., Liu H., Han R. (2016). Molecular evolution and gene expression differences within the HD-Zip Transcription Factor Family of *Zea mays* L.. Genetica.

[20] Kovalchuk N., Chew W., Sornaraj P., Borisjuk N., Yang N., Singh R., Bazanova N., Shavrukov Y., Guendel A., Munz E. (2016). The Homeodomain Transcription Factor TaHD-Zipl-2 from wheat regulates frost tolerance, flowering time and spike development in transgenic barley. New Phytol..

[21] Capella M., Ribone P.A., Arce A.L., Chan R.L. (2015). *Arabidopsis thaliana* HomeoBox 1 (AtHB1), a homedomain-leucine zipper I (HD-Zip I) transcription factor, is regulated by PHYTOCHROME-INTERACTING FACTOR 1 to promote hypocotyl elongation. New Phytol..

[22] Aoyama T., Dong C.H., Wu Y., Carabelli M., Sessa G., Ruberti I., Morelli G., Chua N.H. (1995). Ectopic expression of the *Arabidopsis* transcriptional activator Athb-1 alters leaf cell fate in tobacco. Plant Cell..

[23] Ni Y., Wang X., Li D., Wu Y., Xu W., Li X. (2008). novel cotton homeobox gene and its expression profiling in root development and in response to stresses and phytohormones. Acta Biochim. Biophys. Sin..

[24] Harris J.C., Sornaraj P., Taylor M., Bazanova N., Baumann U., Lovell B., Langridge P., Lopato S., Hrmova M. (2016). Molecular interactions of the γ-clade homeodomain-leucine zipper class I transcription factors during the wheat response to water deficit. Plant Mol. Biol..

[25] Ge X.X., Liu Z., Wu X.M., Chai L.J., Guo W.W. (2015). Genome-wide identification, classification and analysis of HD-Zip gene family in citrus and its potential roles in somatic embryogenesis regulation. Gene.

[26] Song S., Chen Y., Zhao M., Zhang W.H. (2012). A novel *Medicagotruncatula* HD-Zip gene, *MtHB2*, is involved in abiotic stress responses. Environ. Exp. Bot..

[27] Turchi L., Carabelli M., Ruzza V., Possenti M., Sassi M., Peñalosa A., Sessa G., Salvi S., Forte V., Morelli G. (2013). *Arabidopsis* HD-Zip II transcription factors control apical embryo development and meristem function. Development.

[28] Dezar C.A., Giacomelli J.I., Manavella P.A., Ré D.A., Alves-Ferreira M., Baldwin I.T., Bonaventure G., Chan R.L. (2011). HAHB10, a Sunflower HD-ZIP II transcription factor, participates in the induction of flowering and in the control of phytohormone-mediated responses to biotic stress. J. Exp. Bot..

[29] Ooi S.E., Ramli Z., Kulaveerasingam H., Ong-Abdullah M. (2016). *EgHOX1*, a HD-Zip II gene, is highly expressed during early oil palm (*Elaeis guineensis* Jacq.) somatic embryogenesis. Plant Gene.

[30] Franco D.M., Silva E.M., Saldanha L.L., Adachi S.A., Schley T.R., Rodrigues T.M., Dokkedal A.L., Nogueira F.T., Rolim de Almeida L.F. (2015). Flavonoids modify root growth and modulate expression of *SHORT-ROOT* and HD-ZIP III. J. Plant Physiol..

[31] Landau U., Lior A., Leor E.W. (2015). The *ERECTA*, *CLAVATA* and Class III HD-ZIP pathways display synergistic interactions in regulating floral meristem activities. PLoS ONE.

[32] Hawker N.P., Bowman J.L. (2004). Roles for class III HD-Zip and KANADI genes in *Arabidopsis* root development. Plant Physiol..

[33] Zhu Y., Song D., Sun J., Wang X., Li L. (2013). *PtrHB7*, a class III HD-Zip gene, plays a critical role in regulation of vascular cambium differentiation in *Populus*. Mol. Plant..

[34] Carlsbecker A., Lee J.Y., Roberts C.J., Dettmer J., Lehesranta S., Zhou J., Lindgren O., Moreno-Risueno M.A., Vatén A., Thitamadee S. (2010). Cell signalling by microRNA165/6 directs gene dose-dependent root cell fate. Nature.

[35] Li Z., Zhang C., Guo Y., Niu W., Wang Y., Xu Y. (2014). Genome-wide analysis of HD-Zip genes in grape (*Vitis vinifera*). Tree Genet. Genomes..

[36] Kamata N., Okada H., Komeda Y., Takahashi T. (2013). Mutations in epidermis-specific HD-Zip IV genes affect floral organ identity in *Arabidopsis thaliana*. Plant J..

[37] Kamata N., Okada H., Komeda Y., Takahashi T. (2009). The HD-Zip IV Transcription Factor OCL4 is necessary for trichome patterning and anther development in maize. Plant J..

[38] Pan Y., Bo K., Cheng Z., Weng Y. (2015). The loss-of-function *GLABROUS 3* Mutation in Cucumber Is Due to LTR-retrotransposon Insertion in a class IV HD-ZIP transcription factor gene CsGL3 that is epistatic over CsGL1. BMC Plant Biol..

[39] Gill B.S., Appels R., Botha-Oberholster A.M., Buell C.R., Bennetzen J.L., Chalhoub B., Chumley F., Dvorák J., Iwanaga M., Keller B. (2004). A workshop report on wheat genome sequencing. Genetics.

[40] International Wheat Genome Sequencing Consortium (2014). A chromosome-based draft sequence of the hexaploid bread wheat (*Triticum aestivum*) genome. Science.

[41] Kovalchuk N., Wu W., Eini O., Bazanova N., Pallotta M., Shirley N., Singh R., Ismagul A., Eliby S., Johnson A. (2012). The scutellar vascular bundle–specific promoter of the wheat HD-Zip IV transcription factor shows similar spatial and temporal activity in transgenic wheat, barley and rice. Plant Biotechnol. J..

[42] Yang Y., Luang S., Harris J., Riboni M., Li Y., Bazanova N., Hrmova M., Haefele S., Kovalchuk N., Lopato S. (2017). Overexpression of the class I homeodomain transcription factor TaHD-ZipI-5 increases drought and frost tolerance in transgenic wheat. Plant Biotechnol. J..

[43] Kersey P.J., Allen J.E., Armean I., Boddu S., Bolt B.J., Carvalho-Silva D., Christensen M., Davis P., Falin L.J., Grabmueller C. (2015). Ensembl Genomes 2016: More genomes, more complexity. Nucleic Acids Res..

[44] Finn R.D., Mistry J., Schuster-Böckler B., Griffiths-Jones S., Hollich V., Lassmann T., Moxon S., Marshall M., Khanna A., Durbin R. (2006). PFAM: Clans, web tools and services. Nucleic Acids Res..

[45] Wheeler T.J., Eddy S.R. (2013). NHMMER: DNA homology search with profile HMMs. Bioinformatics.

[46] The Conserved Domain Database. https://www.ncbi.nlm.nih.gov/cdd.

[47] Compute pI/mw Tool. https://web.expasy.org/compute_pi/.

[48] Chou K.C., Shen H.B. (2008). Cell-PLoc: A package of web-servers for predicting subcellular localization of proteins in various organisms. Nat. Protoc..

[49] Larkin M., Blackshields G., Brown N., Chenna R., McGettigan P., McWilliam H., Valentin F., Wallace I.M., Wilm A., Lopez R. (2007). Clustal W and Clustal X version 2.0. Bioinformatics.

[50] Tamura K., Stecher G., Peterson D., Filipski A., Kumar S. (2013). MEGA6: Molecular evolutionary genetics analysis version 6.0. Mol. Boil. Evol..

[51] Wang M., Yue H., Feng K., Deng P., Song W., Nie X. (2016). Genome-wide identification, phylogeny and expressional profiles of mitogen activated protein kinase kinasekinase (MAPKKK) gene family in bread wheat (*Triticum aestivum* L.). BMC Genom..

[52] Krzywinski M., Schein J., Birol1 İ., Connors J., Gascoyne R., Horsman D., Jones S.J., Marra M.A. (2009). Circos: An information aesthetic for comparative genomics. Genome Res..

[53] Hu B., Jin J., Guo A.Y., Zhang H., Luo J., Gao Ge. (2015). GSDS 2.0: An upgraded gene feature visualization server. Bioinformatics.

[54] Bailey T.L., Boden M., Buske F.A., Frith M., Grant C.E., Clementi L., Ren J., Li W.W., Noble W.S. (2009). MEME SUITE: Tools for motif discovery and searching. Nucleic Acids Res..

[55] Seq Repository in URGI Wheat Database. https://urgi.versailles.inra.fr/files/RNASeqWheat/.

[56] FastQC. http://www.bioinformatics.babraham.ac.uk/projects/fastqc/.

[57] Trapnell C., Pachter L., Salzberg S.L. (2009). TopHat: Discovering splice junctions with RNA-Seq. Bioinformatics.

[58] Cufflinks 2.2.1. http://cole-trapnell-lab.github.io/cufflinks/releases/v2.2.1/.

[59] Lee T., Yang S., Kim E., Ko Y., Hwang S., Shin J., Shim J.E., Shim H., Kim H., Kim C. (2015). AraNet v2: An improved database of co-functional gene networks for the study of *Arabidopsis thaliana* and 27 other nonmodel plant species. Nucleic Acids Res..

[60] Maere S., Heymans K., Kuiper M. (2005). BiNGO: A Cytoscape plugin to assess overrepresentation of gene ontology categories in biological networks. Bioinformatics.

[61] Primer Premier: A Comprehensive PCR Primer Design Software. http://www.premierbiosoft.com/primerdesign/.

[62] Xu L., Tang Y., Gao S., Su S., Hong L., Wang W., Fang Z., Li X., Ma J., Quan W. (2016). Comprehensive analyses of the annexin gene family in wheat. BMC Genom..

[63] Qiao L., Zhang X., Han X., Zhang L., Li X., Zhan H., Ma J., Luo P., Zhang W., Cui L. (2015). A genome-wide analysis of the *auxin/indole-3-acetic* acid gene family in hexaploid bread wheat (*Triticum aestivum* L.). Front. Plant Sci..

[64] Belamkar V., Weeks N.T., Bharti A.K., Farmer A.D., Graham M.A., Cannon S.B. (2014). Comprehensive characterization and RNA-Seq Profiling of the HD-Zip transcription factor family in soybean (*Glycine max*) during dehydration and salt stress. BMC Genom..

[65] Zhao Y., Zhou Y., Jiang H., Li X., Gan D., Peng X., Zhu S., Cheng B. (2011). Systematic analysis of sequences and expression patterns of drought-responsive members of the HD-Zip gene family in maize. PLoS ONE.

[66] Song A., Li P., Xin J., Chen S., Zhao K., Wu D., Fan Q., Gao T., Chen F., Guan Z. (2016). Transcriptome-wide survey and expression profile analysis of putative chrysanthemum HD-Zip I and II genes. Genes.

[67] Jiang H.Y., Jing J., Huan L., Qing D., Hanwei Y., Defang G., Wei Z., Suwen Z. (2015). Genome-wide analysis of HD-Zip genes in grape (*Vitis vinifera*). Tree Genet. Genom..

[68] Zhang J.Z. (2003). Evolution by gene duplication: An update. Trends Ecol. Evol..

[69] Feldman M., Levy A.A. (2005). Allopolyploidy—A shaping force in the evolution of wheat genomes. Cytogenet. Genome Res..

[70] Lynch M., Force A. (2000). The probability of duplicate gene preservation by subfunctionalization. Genetics.

[71] Ré D.A., Capella M., Bonaventure G., Chan R.L. (2014). Arabidopsis *AtHB7* and *AtHB12* evolved divergently to fine tune processes associated with growth and responses to water stress. BMC Plant Biol..

[72] Yue H., Wang M., Liu S., Du X., Song W., Nie X. (2016). Transcriptome-wide identification and expression profiles of the WRKY transcription factor family in Broomcorn millet (*Panicum miliaceum* L.). BMC Genom..

[73] Schena M., Davis R.W. (1994). structure of homeobox-leucine zipper genes suggests a model for the evolution of gene families. Proc. Natl. Acad. Sci. USA.

[74] Zhang S., Haider I., Kohlen W., Jiang L., Bouwmeester H., Meijer A.H., Schluepmann H., Liu C.M., Ouwerkerk P.B. (2012). Function of the HD-Zip I gene *Oshox22* in ABA-mediated drought and salt tolerances in rice. Plant Mol. Biol..

